# Protective Effect of Polyphenolic Extracts from *Hippophae rhamnoides* L. and *Reynoutria japonica Houtt.* on Erythrocyte Membrane

**DOI:** 10.3390/molecules29133090

**Published:** 2024-06-28

**Authors:** Teresa Kaźmierczak, Katarzyna Męczarska, Sabina Lachowicz-Wiśniewska, Sylwia Cyboran-Mikołajczyk, Jan Oszmiański, Dorota Bonarska-Kujawa

**Affiliations:** 1 Department of Physics and Biophysics, The Faculty of Biotechnology and Food Sciences, Wrocław University of Environmental and Life Sciences, Norwida Str. 25, 50-375 Wrocław, Poland; 2 Faculty of Medicine and Health Sciences, Calisia University, 62-800 Kalisz, Poland; 3 Departament of Fruit, Vegetable and Plant Nutraceutical Technology, The Faculty of Biotechnology and Food Sciences, Wrocław University of Environmental and Life Sciences, Chełmońskiego Str. 37, 51-630 Wroclaw, Poland

**Keywords:** plant extracts, erythrocytes, sea buckthorn, Japanese knotweed, antioxidant activity, hemolysis, fluorimetry, spectrophotometry, UPLC-PDA-ESI-MS/MS

## Abstract

Sea buckthorn and Japanese knotweed are known in many traditional medicine systems to be a great source of bioactive substances. This research aims to compare the bioactivity and protective effects of the phenolic extracts of leaves from sea buckthorn and roots and leaves from the Japanese knotweed on erythrocytes. The polyphenol composition of the extract was analyzed using UPLC-PDA-ESI-MS/MS. The extracts’ toxicity and impact on the erythrocytes’ osmotic fragility were measured spectrophotometrically. The antioxidant activity was determined based on the inhibition of oxidation of erythrocytes and their membrane induced by 2,2′-Azobis(2-methylpropionamidine) dihydrochloride (AAPH),measured spectrophotometrically and using fluorimetry. To find the possible mechanism of the extracts’ action, extract-modified cells were observed under a microscope, and the potential localization of the extract’s phytochemical composition was checked using fluorescent probes. The results showed that the used extracts are not toxic to erythrocytes, increase their osmotic resistance, and successfully protect them against free radicals. Extract components localize on the outer part of the membrane, where they can scavenge the free radicals from the environment. Altogether, the presented extracts can greatly protect living organisms against free radicals and can be used to support the treatment of diseases caused by excess free radicals.

## 1. Introduction

Along with technological progress in the world, we observe a tremendous increase in the number of people with non-communicable diseases (NCDs), known as civilizational or chronic illnesses, e.g., diabetes, cardiovascular and lung diseases, and cancer. Many environmental factors such as high-processed food, air and water pollution, high stress, no body movement, and addictions lead to the disruption of the organism’s homeostasis and, eventually, to illness. According to the World Health Organization, 74% of the yearly global deaths are caused by NCDs, of which cardiovascular diseases constitute the largest percentage [1]. To reduce such a negative increase, many studies have focused on the development of preventative substances. However, synthetical drugs may also harm the organism, so people are looking into more conventional solutions.

Together with the discovery of new synthetic drugs, there is an increasing trend of finding medicinal substances derived from natural sources. Many medical plants that have been known for centuries are a great source of biologically active substances with health benefits and have been used in many traditional medicine systems around the world, including Chinese Medicine. Plants are packed with easily bioavailable nutrients, such as minerals and vitamins, and contain lots of antioxidants, anti-inflammatory, and antimicrobial substances, of which polyphenols constitute the largest group [2]. Many substances are directly derived and purified from plants. There is also a huge trend of using extracts, which contain lots of healthy substances. Although extracts from the same plants have similar health potentials, they may vary in composition according to the cultivation method and the extraction procedure [3].

In this work, two plant extracts, sea buckthorn (*Hippophae rhamnoides* L.) and Japanese knotweed (*Reynoutria japonica Houtt.*) were tested. Both of these plants are used in Chinese medicine in the treatment of several diseases [4,5]. Sea buckthorn is a bush naturally distributed in Asia and Europe. It belongs to the *Eleagnaceae* family, consisting of several species, of which *Hippophae rhamnoides* L. is the most common [6,7]. This plant grows orange berries, which are packed with lots of healthy substances, mainly fatty acids, β-carotene, and flavonoids [6,7,8]. However, it was found that the leaves of *H. rhamnoides* L. contain more biologically active substances than the berries [8,9,10]. Sea buckthorn is used in the treatment of cardiovascular diseases [5,10,11], stomach ulcers [5,12], and skin diseases [13,14]. Japanese knotweed is a native plant in the Far East. It belongs to the family of *Polygonaceae* [4,14]. Leaves and roots of knotweed are the most common and richest sources of biologically active compounds [4]. *R. japonica Houtt.* is rich in stilbenes, flavonoids, anthraquinones, coumarins, and essential oils, which possess high antioxidant activity [3,4]. Extracts of Japanese knotweed are used to treat several illnesses, such as cardiovascular diseases [15,16], cancer [17,18], and neuronal diseases [19,20].

Oxidative stress is the main reason for the development of chronic diseases [21]. Any substance, before getting into the cell, first interacts with the biological membrane [22]. Erythrocytes are the most exposed to the environment, especially oxidants because their main function is to transport oxygen within organisms. Moreover, they interact with other substances in the blood vessels, including those with health benefits.

This research aims to investigate the composition of leaf extract from the sea buckthorn (*Hippophae rhamnoides* L.) (BE), as well as root (RE) and leaf (LE) extracts from the Japanese knotweed (*Reynoutria japonica Houtt.*). Extracts were tested for their protective effects on red blood cells and their isolated membranes against environment-induced oxidative stress. Finally, we tried to rule out the possible mechanism of extract action on the cell membrane. Results from tests are a great comparison of the difference between extracts’ composition and their impact on the protection of red blood cells against oxidation.

Both plants are known for their invasive character [23,24,25]. Buckthorn is used mostly in the production of seed oil and fruit juice [7], and knotweed’s roots have been used for centuries in Japanese cuisine (known as itadori) [25]. The conducted research aimed to expand the current knowledge about the health benefits of their polyphenolic extracts.

## 2. Results

### 2.1. Analysis of Extracts’ Total Phenolic Content (TPC) and Polyphenolic Composition

The total phenolic content (TPC) of the extract was determined, and it was shown that each of the extracts is a rich source of phenolic compounds. Sea buckthorn leaf extract (BE) is the richest source of polyphenols out of all of the used extracts and contains them in the amount of 879.84 ± 3.54 mgGAE/g. Extracts of root (RE) and leaves (LE) of Japanese knotweed also contain lots of phenols, and their content is 445.64 ± 71.74 and 436.55 ± 27.39 mgGAE/g, respectively.

The chromatograms of the prepared extracts, as well as the UV and MS spectra data of polyphenols in all extracts, are presented in Supplementary Materials (Tables S1 and S2, Figures S1 and S2). Chromatograms of extracts vary between plants. It can be seen that sea buckthorn extract (BE) consists of mostly high-molecular compounds, which were eluted from the column very fastly. However, both extracts of the Japanese knotweed (RE and LE) consist of mostly lower molecular substances, which were eluted slower from the column than substances in the BE.

*Hippophae rhamnoides* L. leaf extract (BE) contains 58.26% polyphenols, out of which the largest group are tannins and their derivatives (Table S1). Tannins, such as ellagitannins and hydrolyzable tannins, consist of approx. 48.85% of all polyphenols in extract. In the chromatogram, the highest peaks were obtained for the tannins: chebulagic acid (Rt = 4.32 min; *m*/*z* = 953), ellagitannin (Rt = 5.68, 6.01, 6.31 min; *m*/*z* = 1085), and casuarinin (Rt = 4.32 min; *m*/*z* = 935) (Figure S1). Next, according to the contents, are: flavonols (4.33%) with the highest peak corresponding to isorhamnetin–dihexoside (Rt = 6.90 min, *m*/*z* = 693), phenolic acids (2.4%, e.g., galloyl-bis-HHDP-glucose III, Rt = 5.24 min; *m*/*z* = 935), catechins (1.36%, e.g., catechin–gallocatechin, Rt = 3.70 min; *m*/*z* = 593), different flavonoids (0.82%), and one procyanidin (0.5%)—procyanidin dimmer type B with the Rt = 5.93, *m*/*z* = 577 (Figure S1). The composition of *Reynoutria japonica Houtt*. roots (RE) and leaves (LE) extracts vary significantly (Table S2). However, both contain many polyphenolic compounds, of which RE extract has 40.9% and LE 70.28%. Procyanidins are the largest group of compounds in extracts, accounting for 31.37% in RE and 63.04% in LE. The highest peaks on the RE chromatogram (Figure S2a) correspond to the trans-piceid (Rt = 7.92 min; *m*/*z* = 389) and trans-resveratroloside (Rt = 7.19 min; *m*/*z* = 449), which belong to the stilbenes group. All stilbenes and stilbenoids consist of 6.01% of the RE, but LE contains only 0.09% of them. Furthermore, catechins consist of 3.37% of the RE and only 0.83% of the LE. (−)-Epicatechin corresponds to 2.64% in RE and 0.84% in LE (Rt = 5.61 min; *m*/*z* = 289). Phenolic acids consist of 0.15% RE and 2.23% of LE polyphenols. Only leaf extract contains polyphenols from flavonols (4.09%) (Table S2). The highest peak for LE was quercetin rhamnoside, which belongs to the flavonols (Rt = 9.34 min; *m*/*z* = 447) (Figure S2b).

### 2.2. Determination of the Anti-Radical Activity of the Extracts

The anti-radical activity of the extracts was investigated based on their ability to reduce DPPH radicals. The results are shown in Table 1.

The results showed that the extract causes a significant reduction in DPPH radicals in comparison to the L (+)-ascorbic acid, treated as a standard antioxidant. The best anti-radical activity was exhibited by the root extract of Japanese knotweed (RE) (Table 1).

### 2.3. Hemolytic Activity of Extracts and Their Impact on Osmotic Fragility of Erythrocytes

Hemolytic activity of the extracts and their impact on erythrocytes’ osmotic fragility was determined using a spectrophotometrically measured release of the hemoglobin. The results show that both the sea buckthorn and the Japanese knotweed extracts do not have destructive activity on the erythrocyte membrane in the concentrations used in the experiment.

**Figure 1:**
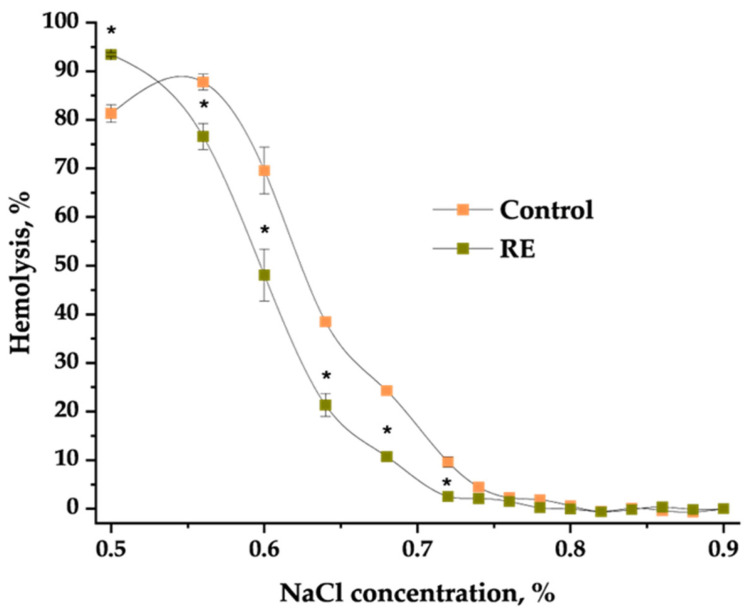
The hemolytic curve for the control cells and cells modified with root extract of Japanese knotweed (RE) in a concentration of 50 µg/mL. Statistically significant differences are marked as * with *p* < 0.05.

However, extracts change the osmotic fragility of the erythrocytes. C_50_ values for each of the extracts were as follows: for control cells—0.65 ± 0.01%NaCl; for BE—0.63 ± 0.1%NaCl; for RE—0.60 ± 0.01%NaCl*; for LE—0.67 ± 0.01%NaCl* (*statistically different values in relation to control, *p* < 0.05). The C_50_ values indicate that the used extracts make erythrocytes more resistant and intact to osmotic pressure than was observed in the control group. Figure 1 shows the hemolytic curves for the control cells modified with 50 µg/mL of RE. The root extract of Japanese knotweed significantly shifts the hemolytic curve towards lower NaCl concentrations compared to the control. The effect was observed only for the higher concentration of the extracts; therefore, a 50 μg/mL concentration was used in this experiment.

### 2.4. Antioxidant Properties of Extracts against Erythrocytes

A series of experiments with oxidation inducer 2,2′-Azobis(2-methylpropionamidine) dihydrochloride (AAPH) were performed to examine the potential antioxidant activity of extracts on erythrocytes and erythrocyte membranes.

Firstly, the antioxidant activity of extracts was checked based on their ability to inhibit AAPH-induced hemolysis of erythrocytes. The results are shown in Table 2 and Figure S3a–c. The ability of the extracts to protect the erythrocyte membranes (ghosts) against oxidative stress was also checked based on the inhibition of oxidation with AAPH. The comparison of the IC_50_ of extracts that caused 50% oxidation inhibition is listed in Table 2.

All the extracts protect red blood cells from oxidative hemolysis. The IC_50_ of all extracts that inhibit hemolysis are as follows: 13.77 ± 3.07 µg/mL (BE), 15.7 ± 1.34 µg/mL (RE), and 6.00 ± 3.02 µg/mL (LE) (Table 2). All results of inhibition varied significantly from the control sample values. The leaf extract of Japanese knotweed has the best protective effect on the whole erythrocyte against AAPH oxidation. All extracts protected the cells in a much better way than the standard antioxidant ascorbic acid (AA) (Table 2).

The results for the antioxidant abilities of extracts on erythrocyte membranes (ghosts) show that all the extracts are successful in the means of oxidation inhibition induced by AAPH. RE showed the best antioxidant activity against AAPH out of all used extracts, with the lowest IC_50_ value (Table 2). In the mean of AAPH-induced oxidation, all extracts had slightly lower antioxidant activity than Trolox^®^, but they still have very comparable activity (Table 2).

### 2.5. Microscopic Observations

**Figure 2:**
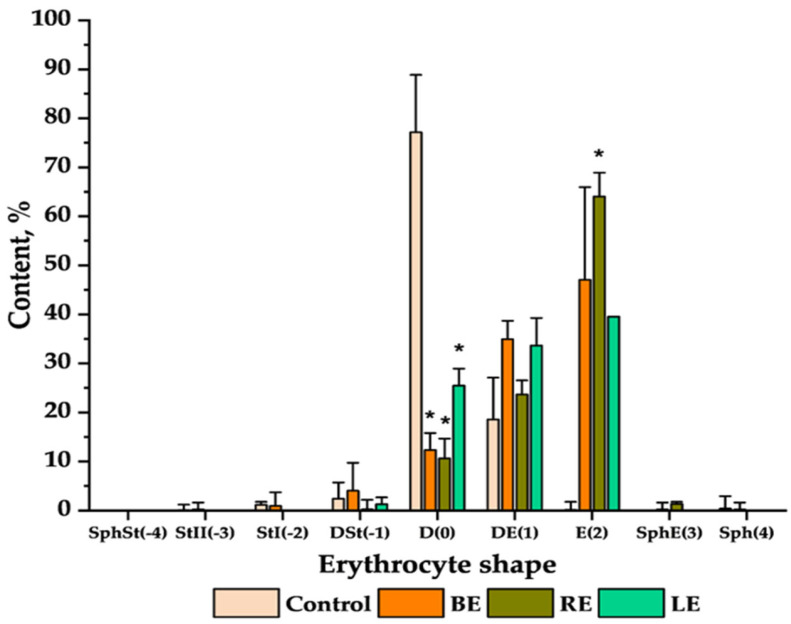
The percentage content of the erythrocyte shapes induced with 50 μg/mL of BE, RE, and LE extracts. The names of shapes and their morphological indexes, written in parentheses, are as follows: spherostomatocytes (SphSt(-4)), second-order stomatocytes (StII(-3)), first-order stomatocytes (StI(-2)), discostomatocytes (DSt(-1)), discocytes (D(0)), discoechinocytes (DE(1)), echinocytes (E(2)), spheroechinocytes (SfE(3)), spherocytes (Sf(4)). Statistically significant differences are marked as * with *p* < 0.05.

To study the ability of extracts to induce erythrocyte shape change, different amounts of the extracts were incubated with erythrocytes and then the cells were observed under a microscope. The percentage content of each of the erythrocyte shapes, according to the morphological indexes of the Bessis scale [28] for control erythrocytes and cells treated with 50 μg/mL of extracts (concentration that showed significant results) is shown in Figure 2. The microscopic images of the control sample and modified erythrocytes are presented in Figure S4 (Supplementary Materials). All the extracts induced the echinocyte shape formation.

### 2.6. Impact of Extracts on the Fluidity of the Erythrocyte Membrane

**Figure 3:**
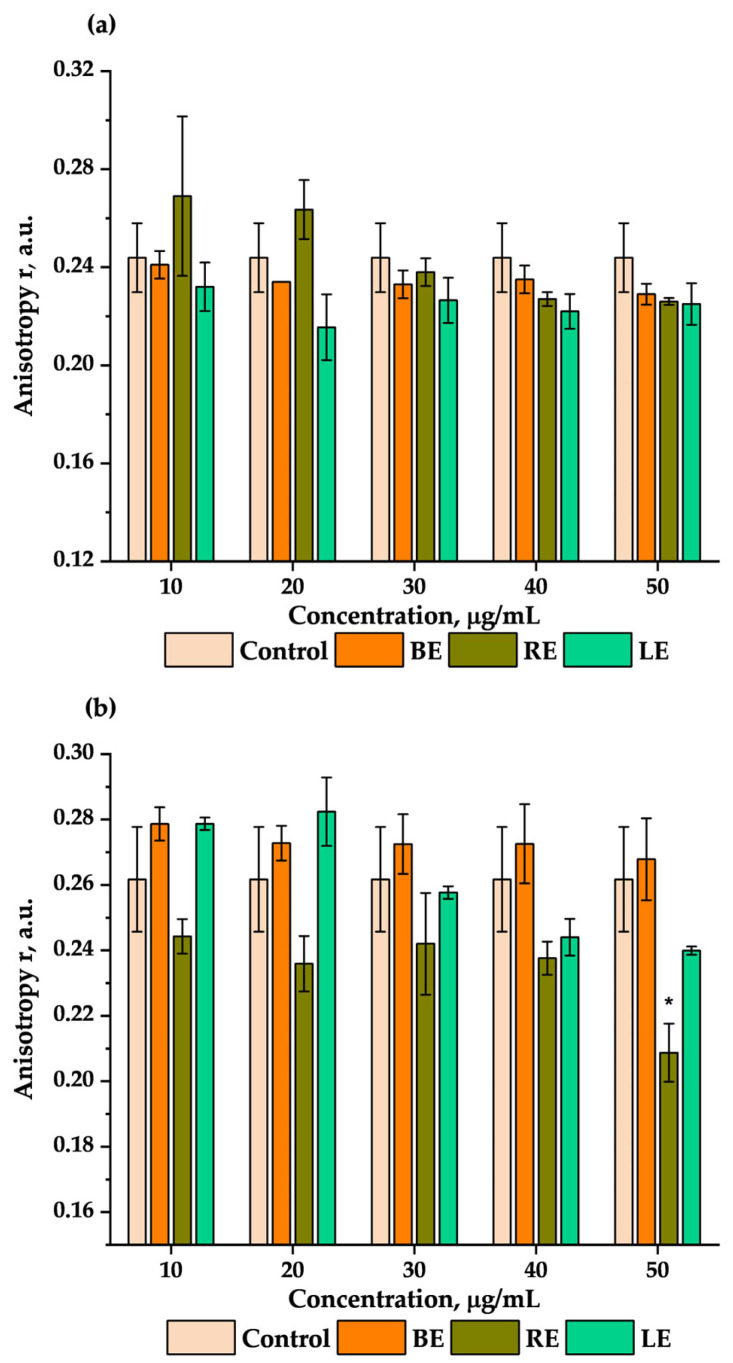
Changes in the anisotropy (r) of the DPH (**a**) and TMA-DPH (**b**) probe with the different concentrations of BE, RE, and LE. Statistically significant differences are marked as * with *p* < 0.05.

To test the impact of the extracts on the fluidity of the isolated membrane of erythrocytes, measurements with fluorescent probes DPH and TMA-DPH were performed. The comparison of the anisotropy changes of the DPH (a) and TMA-DPH (b) probes with different concentrations of the extracts is shown in Figure 3.

For the DPH anisotropy, there were no significant differences between the used concentrations for all the extracts. The anisotropy of the DPH probe with extract-modified samples was at a similar level as control cells (Figure 3a). However, there were small changes in the anisotropy of the TMA-DPH probe for the RE. The highest concentration of RE (50 μg/mL) was statistically significantly lower than the control sample (Figure 3b). This indicates that root extract of Japanese knotweed in higher concentrations contributes to the changes in the membrane fluidity on the level of the TMA-DPH probe localization in the erythrocyte membrane.

## 3. Discussion

Plant extracts are a great and easily available source of bioactive substances that can possess healthy benefits for living organisms. However, to fully comprehend their therapeutic potential, it is crucial to understand their diverse composition and corresponding biological activities. In this research, we prepared and analyzed three polyphenolic extracts derived from the leaves of the sea buckthorn (*Hippophae rhamnoides* L.) as well as the leaves and roots of the Japanese knotweed (*Reynoutria japonica Houtt.*) and tested their bioactivity. Protective effects, such as the antioxidant properties of extracts, were tested on the erythrocytes, as they are mostly exposed to oxidative stress. This study compares the composition and bioactive and protective properties of these extracts.

The extraction was processed to obtain extracts rich in polyphenolic compounds. This was confirmed by results from the UPLC-PDA-ESI-MS/MS and total phenolic content (TPC) analyses. Sea buckthorn extract (BE) is rich in polyphenols, mainly from the group of tannins and their derivatives: hydrolyzable tannins or ellagitannins (Table S1). Tannins are non-flavonoid compounds that are the secondary metabolites of plants and are known for their bitter taste [29]. Although tannins are known to have toxic effects [30,31], they still possess a lot of health benefits, which contribute to their large interest in research areas. Tannins, especially galloyl ones, have antimicrobial activity. They can either bind with the proteins on the bacterial cell wall [32], interact with bacterial enzymes, or directly damage the outer wall and bacterial membrane [33,34,35]. Moreover, tannins have antioxidant properties. Researchers found that they can possess better antioxidant properties than monomeric polyphenols [36]. Our results of polyphenolic composition correspond to some other research. We have found some flavonoid compounds (isorhamnetin isomers, quercetin) in sea buckthorn extract besides tannins (Table S1). This has been also found in another study [37]. Catechin and its derivatives were also detected by other researchers, as described in [38]. Most of the published research mentions the fact that leaves of sea buckthorn contain lots of gallic acid [39]. Our research did not show the presence of gallic acid in the chromatogram (Figure S1). A study conducted by Heinäaho, Pusenius, and Julkunen-Tiitto also mention that leaves of *H. rhamnoides* L. contain a large number of tannins, which is in accordance with our obtained results [40]. Extracts from the Japanese knotweed vary drastically in order of their composition (Table S2). However, procyanidins constitute the largest percentage of the content of both leaf and root extracts (Table S2). Procyanidins are the polymers of catechins [41]. They can scavenge free radicals [42,43], regenerate antioxidants such as ascorbic acid [44], chelate pro-oxidant transition metals [4,7,45,46], and protect organisms against UV radiation [45,47]. RE contains stilbenes in a much higher percentage than LE (Table S2). Stilbenes, such as resveratrol and piceid, are non-flavonoid compounds that have many of the healthy properties, including anticancer activities [17,18]. Our results also showed that the root of Japanese knotweed contains some catechins, particularly (−)-epicatechin, which has also been shown in another work [3,48] (Table S2). Another work also showed that roots of *R. japonica Houtt.* contain stilbenoids, such as resveratrol [49], which corresponds to our research. The other study showed that the roots and leaves of Japanese knotweed are rich in proanthocyanidins [50]. We have also shown that both roots and leaves of this plant contain lots of polymeric proanthocyanidins—protoanthocyanidins. In RE, they consist of 31.37% and in leaves—63.04% (Table S2). The other compounds that were not mentioned in the analysis belong to the high-weight polyphenols, which have antioxidant and health benefits and contribute to the extracts’ bioactivity.

The Folin-Ciocâlteu (F-C) assay is a common and simple method used in the many fields of science and industry. It can be used to measure not only the total phenolic composition (TPC) but also the reducing power of the extracts and beverages [51]. It is based on the reaction of an electron transfer between an antioxidant (polyphenol) and F-C reagent [52]. The reduction of the acids by antioxidant reagents produces a change in the solutions’ color, which has maximum absorbance at 765 nm [51,52,53]. It is worth mentioning that assays based on single electron transfer (SET), such as F-C, DPPH, or FRAP assays, strongly correlate with the structure of the compounds [51,52]. Bors proposed criteria for the antioxidant properties of the phenolic compounds: (1) the presence of the catechol group in the B-ring, (2) a double bond between the second and third atoms combined with the 4-oxo group in the C-ring, and (3) the presence of hydroxyl groups at positions 3 and 5 combined with 4-oxo groups [54,55]. F-C assay results can be easily affected by other reducing components such as sugars and ascorbic acid [56,57,58]. In this investigation, the other components, besides polyphenols, were eliminated from the extracts during the purification process. Therefore, the F-C assay shows the real polyphenolic content and the reducing power of the phenolic compounds. According to the research, sea buckthorn extract (BE) contains the highest number of polyphenolic compounds (879.84 ± 3.54 mgGAE/g). It might be attributed to the high concentration of tannins (Tables S1 and S2), which have many hydroxyl groups in their structure [43]. Consequently, it might be concluded that this extract possesses the highest antioxidant activity out of all extracts in this research. However, the antioxidant properties of compounds are not only based on the electron transfer between a substance and radicals. Many other mechanisms, including their role in the antioxidant cellular mechanisms, are important and mentioned further in the discussion.

In order to expand the possibility of finding the extracts’ mechanisms of action, additional research using the DPPH radical was conducted. 2,2-diphenyl-1-picrylhydrazyl (DPPH) is a stable chromogenic radical, and after a reduction by the electron transfer from another substance (antioxidant) to the impaired electron in the molecule, a decrease in its absorption at 517 nm is observed [59]. The reaction mechanisms are a single electron transfer (SET) and hydrogen atom transfer (HAT) [59]. Here, the results showed that all the extracts significantly reduced DPPH radicals by 50% with the concentrations of 6.28 ± 0.06 μg/mL (BE), 5.89 ± 0.47 μg/mL (RE), and 6.03 ± 0.65 μg/mL (LE) (Table 1). The best anti-radical activity was found with the root extract of the Japanese knotweed, but it had statistically comparable effectiveness to the leaf extract. The comparable effect of Japanese knotweed extracts can be attributed to their very similar TFC and high content of procyanidins. However, RE contains a bit more catechins (Table S2), which are known for their great ability to reduce the DPPH radical, confirmed by other researchers [60,61]. However, we found that extract of the sea buckthorn leaves reduced the DPPH radical in the lowest concentration of 15 μg/mL by 94.44 ± 0.55% in comparison to the RE (92.70 ± 0.71%) and LE (88.09 ± 0.63%). Therefore, because of the highest content of tannins (Table S1), they reduced the DPPH radicals more efficiently in the lower concentrations. Overall, extracts have shown much better anti-radical activity than standard antioxidant L (+)-ascorbic acid, which has less anti-radical activity [62]. Other researchers also confirmed that extracts have high anti-radical activity based on DPPH reduction activity [3,48,63,64].

It is important to know that even if the extract contains lots of compounds with health benefits, it may possess some toxicity toward the cells. Therefore, the toxicity effects of BE, RE, and LE were studied on the erythrocytes. The results show that all the extracts in concentrations between 1 and 100 µg/mL do not act destructively on the erythrocyte membrane. Consequently, there are no contraindications to the use of extracts for the next tests in higher concentrations as they are safe. It was found that crude extract from the leaves of sea buckthorn did not induce erythrocyte hemolysis, even in higher concentrations [63]. Japanese knotweed root extract has been shown to have anticancer properties, as it was cytotoxic to the cancer cells but also inhibited the growth of some infectious bacteria and yeasts [3]. There are a scarce number of publications that mention the effect of Japanese knotweed extracts on erythrocytes in vitro. However, Kovářová et al. showed that the supplementation of Japanese knotweed in the horse diet significantly improves the quality of blood in horses, i.e., decreases the level of cholesterol [65].

The interaction of extracts with the erythrocyte membrane components, such as membrane proteins, contributes to the shape and stability of change in the sensitivity of erythrocytes to the external environment [66,67]. It was found that erythrocytes in pathogenic stages, observed, for example, in anemia or cancer, are more fragile than normal healthy cells [9,66,68]. Therefore, it is important to find a medicinal substance that does not decrease the resistance of the erythrocyte membrane. The impact of the extracts on the erythrocytes’ osmotic fragility in the hypotonic solution was checked. Hemolytic curves for BE and RE in concentrations of 50 µg/mL are shifted toward lower NaCl concentrations in comparison to the control cells. However, only RE in the highest concentration gives a statistically significant change in C_50_ following the control cells (Figure 1). These results confirm that extracts, especially RE, modify erythrocytes to be more resistant to the hemolysis induced by the hypotonic environments. For BE, it can be concluded that the higher concentrations of tannins may be responsible for this effect, as confirmed by other researchers who studied the osmotic fragility effects of the sumac extracts rich in tannins [69]. The results for the resistant effect of RE on erythrocytes do not have any comparisons in the literature to this day. However, it was found that grape extract, rich in procyanidins, possessed a healthy impact on the erythrocytes and had an anti-hemolytic effect [70].

Extracts used in this research are a great source of polyphenolic compounds that have documented antioxidant activities alone. However, there is a scarce amount of research on the extracts in accord with the protection from the environmental stress of red blood cells. Therefore, we conducted experiments to check if BE, RE, and LE have antioxidant properties and can protect the erythrocytes against environmental stress.

2,2′-azobis (2-amidinopropane) dihydrochloride (AAPH) is a commonly used oxidant. It induces the formation of free radicals through spontaneous decomposition at 37 °C [71]. Then, the radicals react with oxygen and cause oxidation of the lipids [63]. We used AAPH to check the ability of the extracts to protect the erythrocytes from the generated free radicals, measured by the inhibition of induced hemolysis. The results showed that all the extracts protect red blood cells against AAPH-induced hemolysis (Figure S3a–c). All the results are comparable; however, the leaf extract of Japanese knotweed (LE) had a slightly better effect than the other, as its IC_50_ was 6.00 ± 3.02 µM (Table 2). The great ability of this extract to inhibit the oxidation of erythrocytes is due to the high phenolic content and the polyphenols consisting of 70.28% of the extract (Table S2). Procyanidins, which consist of 63.04% of all extract, contribute to the high antioxidant activity and protect the erythrocytes against induced oxidative stress. However, it inhibited induced hemolysis in the lower concentration, but the highest inhibition was 55.80 ± 8.87% (15 µg/mL) (Figure S3c). On the other hand, sea buckthorn extract (BE) had IC_50_ 13.77 ± 3.07 µg/mL, but the inhibition went to 74.43 ± 8.00% for 15 µg/mL and even 96.12 ± 1.08% for the concentration of 50 µg/mL (Figure S3a). These results show that BE, with the highest TFC of 879.84 ± 3.54 mgGAE/g and the highest percentage of tannins, protects erythrocytes much better than RE and LE against AAPH-induced hemolysis. This might correspond to the Bors criteria and high level of tannins in the extract [52,54], but also the impact on the cellular antioxidant systems. The extracts protected the cells much better than the standard antioxidant—ascorbic acid (AA) (Table 2). Therefore, it can be concluded that they are safer and much better antioxidants against AAPH-induced hemolysis.

To investigate the protective effect of extracts on the erythrocyte membrane, oxidation inhibition tests on the isolated ghosts were performed using AAPH oxidant. The best results for the inhibition of the AAPH-induced oxidation of the erythrocyte membrane were the root extract of Japanese knotweed (RE), which had IC_50_ of 6.42 ± 0.68 μg/mL (Table 2). That is a surprising result because, as stated above, it protected the erythrocytes from the AAPH-induced hemolysis at the lowest level of all extracts. The highest protective effect of this extract can be attributed to the certain amount of catechins, stilbenes, and procyanidins (Table S2). They all possess antioxidant effects, which can work synergically to give the best outcome. Moreover, it was found that grape extract rich in procyanidins such as RE can contribute to the increase in Cu/Zn-superoxide activity [72]. According to that, it could be concluded that extracts can not only possess an antioxidant activity of free radical scavenging but may also induce many of the cellular mechanisms that combat oxidative stress. BE and LE extracts had comparable protective activity against two of the inductors. All in all, extracts inhibited the oxidation of the erythrocyte membrane at a very high level, as is seen by the very low IC_50_ values (Table 2).

The protective effect of BE, RE, and LE can be attributed to their interactions with the cell membrane. Thus, the changes in the erythrocyte shapes after modifications with extracts were observed under the microscope. Polyphenols possess an amphipathic nature, and they can localize in the different parts of cell membranes, where they can change in shape [28,73]. Erythrocytes, modified with extracts, are more echinocytic, following the Bessis scale (Figure 2 and Figure S4) [28]. This confirms that extracts have a better ability to localize on the erythrocyte’s surface rather than in the inner parts of the lipid membrane [73,74,75]. Another argument that confirms it is that extracts have more hydrophilic features, and they can mostly interact with the outer layer of the cell membrane. The reason BE induces the slight spherocyte formation (Figure 2) is due to the high interactions with the surface of the erythrocyte, causing the echinocytes to lose their spikes [73]. However, it was confirmed that hydrolyzable tannins and ellagitannins, which consist of a higher percentage of BE content, are more hydrophobic, especially with the additional galloyl or HHDP groups (Table S1) [76]. On the other hand, Japanese knotweed extracts contributed to the formation of echinocytes and slight spherocytes (Figure 2). These results show that RE and LE are more hydrophilic and interact mostly with the outer layer of the membrane. Procyanidins, which are the main components of the knotweed extracts (Table S2), are characterized by the higher number of hydroxyl groups in the structures, and their isolation from the extracts is mostly by hydrophilic interaction chromatography (HILIC) [77]. Thus, it can be speculated that the induction of the echinocyte shapes by those extracts is attributed to the hydrophilic character of these compounds.

To check and confirm the influence of the extracts on the outer layer of the erythrocyte membrane, fluorimetric measurements using fluorescent probes that localize on different levels of the lipid membrane were conducted. The DPH probe localizes deep in the lipid membrane on the level of carbohydrate chains [78]. The results show that the extracts did not change the anisotropy of the DPH probe in comparison with the control sample (Figure 3a). Therefore, it can be concluded that the three extracts do not interact with the hydrophobic parts of the erythrocytes’ lipid membrane and that they do not change their fluidity. The next probe that was used in the experiment was TMA-DPH, which localizes at the level of the fourth carbon atom in the fatty acid chain. Neither BE nor LE changes the anisotropy of the TMA-DPH probe (Figure 3b). However, RE in the highest concentration significantly decreased the anisotropy of TMA-DPH relative to the control sample (Figure 3b). Changes in the anisotropy of TMA-DPH, caused by the RE, contribute to the possible intercalation of this probe into this part of the membrane. The decrease in the anisotropy causes this part of the membrane to be more fluid than in control cells [79]. These results can be compared with another study, which found that procyanidin B_3_ interacts with both hydrophobic and hydrophilic parts of the membrane [80].

All the extracts possessed high antioxidant activity. They protected the erythrocyte membrane against AAPH-induced oxidation. The possible mechanism of the protection of the membrane is that BE, RE, and LE localize to the hydrophilic part of the cell membrane. In this area, they protect the cells from environmental stress, such as free radicals, and induce other cellular or serum mechanisms, which combat oxidative stress. More studies will be conducted to dive more into interactions of the extracts with other blood cells in order to find their mechanisms of action and prevention of diseases.

## 4. Materials and Methods

### 4.1. Materials

Heparin was bought in Polfa Warszawa (Warszawa, Poland). Substances used for the preparation of buffers were NaCl (Avantor Performance Materials, Gliwice, Poland), Na_2_HPO_4_·H_2_O, NaH_2_PO_4_·H_2_O, EDTA, Tris (Chempur, Piekary Śląskie, Poland). 2,2′-Azobis(2-methylpropionamidine) dihydrochloride (AAPH), fluorescent probes (DPH, TMA-DPH), dimethylformamide (DMF) for dissolving probes, Follin–Ciocâlteu reagent, 2,2-diphenyl-1-picrylhydrazyl (DPPH), L (+)-ascorbic acid, glutaraldehyde, formic acid, acetonitrile, leucine enkephalin were bought in Merck (Dramstadt, Germany). Ethanol was bought from Avantor Performance Materials, Gliwice, Poland. Thiobarbituric acid (TBA) and immersion oil were bought in Honeywell Fluka (Charlotte, NC, USA), and trichloroacetic acid (TCA) was bought in Eurochem BGD (Tarnów, Poland).

### 4.2. Preparation of Plant Material

The *Reynoutria japonica Houtt.* roots and leaves (each 100 g) and *Hippophae rhamnoides* L. leaves (100 g) were harvested from the Garden of Medicinal Plants herbarium of the Medical University in Wrocław, Poland, by cultivation in the University’s experimental field. Following the harvest, the specimens were promptly subjected to cryopreservation in liquid nitrogen and subsequently freeze-dried for a duration of 24 h (Labconco Corporation freeze dryer, Kansas City, MO, USA). Homogeneous powdered samples were then obtained by pulverizing the desiccated tissues within a closed laboratory mill to prevent hydration. These powders were stored in a refrigerator at a temperature of −80 °C until they were utilized for extract preparation. The polyphenol extraction protocol was previously detailed [81]. Polyphenols were extracted using water containing 200 ppm SO_2_, with a solvent-to-leaves ratio of 3:1 (*v*/*v*). Subsequently, the extract was adsorbed onto Purolite AP 400 resin (Purolite™, Ecolab, King of Prussia, PA, USA) for additional purification. The isolated polyphenols were then eluted with 80% ethanol, concentrated, and subjected to freeze-drying. 

### 4.3. Analysis of Extracts by UPLC-PDA-ESI-MS/MS

Polyphenols within the extracts were characterized utilizing an ACQUITY Ultra Performance LCTM system (UPLC) featuring a binary solvent manager (Waters, Milford, MA, USA) coupled with a Micromass Q-Tof micro–mass spectrometer (Waters, Manchester, UK), equipped with an electrospray ionization (ESI) source capable of operating in both negative and positive ionization modes. MassLynxTM software (version 4.1; Waters, Milford, MA, USA) facilitated instrument control, data acquisition, and processing. Individual polyphenols underwent separation employing a UPLC BEH C18 column (1.7 μm, 2.1 × 50 mm; Waters, Milford, MA, USA) maintained at 30 °C. A sample volume of 10 μL was injected, and elution was accomplished over 15 min utilizing a combination of linear gradients and isocratic flow rates set at 0.45 mL/min. The mobile phase consisted of solvent A (1.5% formic acid, *v*/*v*) and solvent B (100% acetonitrile). The elution program commenced with an isocratic phase of 99% A (0–1 min), followed by a linear gradient reaching 0% A over 12 min; from 12.5 to 13.5 min, the system reverted to the initial composition (99% A) and was subsequently held constant for column re-equilibration. Full-scan, data-dependent MS scanning spanning 100 to 1500 *m*/*z* was employed for analysis, with a mass tolerance of 0.001 Daltons and a resolution of 5000. Leucine enkephalin served as the internal reference compound for ESI-MS accurate mass experiments, introduced via the LockSpray channel using a Hamilton pump. The lock mass correction was set at ±1000 for Mass Window. Time-of-flight MS chromatograms were presented as base peak intensity chromatograms and normalized to 12,400 counts per second (=100%). The effluent was directed to the electrospray source, featuring a source block temperature of 130 °C, a desolvation temperature of 350 °C, a capillary voltage of 2.5 kV, and a cone voltage of 30 V. Nitrogen gas served as the desolvation gas at a flow rate of 300 L/h. Subsequent to retention time alignment and accurate molecular mass determination, individual components were optimized to their estimated molecular mass [M-H] in both negative and positive ionization modes pre- and post-fragmentation. UPLC-MS data were then imported into MassLynx 4.0 ChromaLynxTM Application Manager software for further analysis, allowing for the identification of characterized substances across different samples.

### 4.4. Determination of Total Phenolic Content (TPC) in Extracts

Extracts were dissolved in the phosphate buffer (100 mL of 0.103 M Na_2_HPO_4_·H_2_O and 12 mL of 0.154 M NaH_2_PO_4_·H_2_O) to obtain concentrations between 1 and 10 mg/mL. Total phenolic content (TPC) in extracts was determined by the method using Follin–Ciocâlteu reagent [53,82]. The standard curve was determined based on gallic acid. The results were expressed as mg gallic acid equivalents (GAE) per 1 g of dry sample (mgGAE/g).

### 4.5. Investigation of the Anti-Radical Activity of Extracts

To determine the ability of extract to reduce free radicals, the assay using 2,2-diphenyl-1-picrylhydrazyl (DPPH) radical was used. The research was conducted based on the previous research posted by Gerçek et al. after modifications [83]. The procedure was conducted using a 96-well microplate. Briefly, DPPH was dissolved in ethanol to obtain a concentration of 200 μM; therefore, after the addition of 200 μL of the DPPH solution to the well, the absorbance was around 1. To each well, the extracts dissolved in the water were added in concentrations ranging from 1 to 100 μg/mL. Next, DPPH was added, and the plate was incubated while constantly mixing for 30 min in a dark place. Instantly, the absorption of the solution in each well was measured using Epoch Microplate Spectrophotometer (BioTek Instruments, Winooski, VT, USA). The wavelength was set at 517 nm. The blank sample constituted pure ethanol.

The percentage of the DPPH radical reduction by concentration of each extract was calculated using the Formula (1):

(1) $$\%\;reduction=\frac{A_{C}-A_{S}}{A_{C}}\times 100\%$$

where A_C_—absorbance of the control (DPPH), A_S_—absorbance of the sample (DPPH with extracts). Consequently, based on the slope, the effective concentrations of the extract that caused 50% of the reduction of the DPPH radical (EC_50_) were counted. The results were compared with the standard antioxidant–L (+)-ascorbic acid.

### 4.6. Preparation of Erythrocytes

Experiments were performed using fresh, heparinized pig blood. The choice of pig blood resulted from the fact that the lipid composition of pig erythrocyte membranes is similar to human erythrocytes [74], and the blood itself is very easily accessible. Pig blood was bought in the slaughterhouse. According to Polish law, permission from the ethics committee to use pig blood for the experiments is not necessary. Blood was prepared according to the procedure described previously by Cyboran-Mikołajczyk et al. [84] after modification. Briefly, after the removal of the plasma by centrifugation at 4 °C, 2500 rpm, erythrocytes were washed a minimum 3 times with PBS 310 mOsm pH 7.4 (100 mL phosphate buffer, 1 L 0.9% NaCl and 0.1 M EDTA) to obtain clear supernatant. The next steps of the blood preparation were conducted according to the following procedures.

### 4.7. Preparation of Erythrocyte Membranes (Ghosts)

To obtain erythrocyte membranes from pig blood, the Dodge method was used [85]. The method uses the osmosis effect, by which hemoglobin is removed from the erythrocytes, leaving only the outer membrane of the cell. To each 1 mL of blood, 14 mL of hemolytic buffer PBS 20 mOsm (100 mL phosphate buffer, 1434.5 mL distilled water, and 15.5 mL 0.1 M EDTA) was added, and the mixture was incubated for 1 h in 4 °C and then centrifuged in 4 °C, 12,000 rpm. After that, the supernatant was removed, and membranes were washed at least 3 times with PBS 20 mOsm to obtain clear membrane suspension. Membranes were suspended in PBS 20 mOsm to obtain a protein concentration of around 100 mg/mL, measured using the Bradford method [86].

### 4.8. Hemolytic Activity of Extracts and Their Impact on the Erythrocytes’ Osmotic Resistance

To obtain information about the toxicity of each extract, hemolytic tests were run [79] on fresh blood modified with different concentrations of BE, RE, and LE (1–100 µg/mL). The absorbance of released hemoglobin from the blood cells was measured in λ = 540 nm, using a Cary 300 Bio UV-Visible Spectrophotometer (Agilent Technologies, Santa Clara, CA, USA). The % of hemolysis was the feature of the extract’s cytotoxicity. The osmotic resistance test was prepared using a similar spectroscopic method [79]. The concentration of extracts used was 50 μg/mL. The percentages of hemolysis vs. NaCl concentrations were used to prepare the hemolytic curve. Then, C_50_, the NaCl concentration that causes 50% of erythrocyte hemolysis, was determined.

### 4.9. Antioxidant Activity of Extracts

#### 4.9.1. Protection of Erythrocytes against AAPH

The method to check the protection of erythrocytes against AAPH oxidizing agents was developed previously by Męczarska et al. [87]. Blood samples were incubated with different concentrations of extracts from 5 to 25 µg/mL for 1 h at 37 °C. Next, 120 µL 1 M AAPH (dissolved in distilled water at 37 °C to obtain a clear solution) was added, and samples were incubated for 1 h. To the samples, 2 mL phosphate buffer was added, and samples were centrifuged for 15 min, 2500 rpm. Finally, the absorbance of the released hemoglobin was measured in λ = 540 nm, and the % of inhibition was counted using the following formula:

(2) $$\%inhibition=\frac{\%Hc-\%Hs}{\%Hc}\times 100\%$$

where %Hc is the percent of hemolysis for the control sample, and %Hs is the percent of hemolysis of the extract-modified sample.

Based on the percentage of inhibition, IC_50_ for each extract was determined.

#### 4.9.2. Protection of Erythrocyte Membranes against AAPH

The method using the changes in the fluorescence of the TMA-DPH probe in the ghosts modified with extracts was developed previously in [27,88]. The fluorescence of the TMA-DPH probe in the ghosts incubated with different concentrations of the extracts was measured for 30 min at 37 °C, using the Varian Cary Eclipse Spectrofluorometer with the addition of thermal block Varian SPVF. The excitation and emission wavelengths of the TMA-DPH probe were 360 and 430 nm, respectively.

Oxidation of the lipids in erythrocyte membranes was calculated using relative fluorescence as the rate of fluorescence of the TMA-DPH probe oxidized with AAPH to the beginning fluorescence of the probe. The percent of oxidizing inhibition by extracts was counted based on the following equation:

(3) $$\%inhibition=\frac{F_{s}-F_{c}}{F_{r}-F_{c}}\times 100\%$$

where F_s_—is the fluorescence intensity of the sample with extract and AAPH after 30 min, F_c_—is the fluorescence intensity of the control sample with AAPH after 30 min, and F_r_—the relative fluorescence intensity of the reference sample after 30 min.

### 4.10. Microscopic Preparations

Microscopic observations were used to check the ability of extracts to modify the membrane of erythrocytes [73,74,75]. Blood samples, suspended in NaCl and with 10 and 50 µg/mL of extracts, were incubated for 1 h at 37 °C. Next, to preserve erythrocyte shapes, 2 µL 2.5% glutaraldehyde was added to each sample, and they were incubated for 30 min at room temperature. Microscopic observations were conducted on the Nikon ECLIPSE E200 (Nikon Europe B.V., Amstelveen, The Netherlands) with the camera attached to the microscope (MOTICAM S6) (magnification 1000×) [27].

### 4.11. Changes in the Fluidity of the Membrane

In this experiment, two fluorescent probes: 1,6-diphenyl-1,3,5-hexatriene (DPH) and 1-(4-Trimethylammoniumphenyl)-6-Phenyl-1,3,5-Hexatriene p-toluenesulfonate (TMA-DPH) were used. They all localize in different parts of the membrane. Changes in the fluorescence of each probe can determine the changes in the fluidity of a particular membrane area and can give information on the potential localization of extract components.

Probes and samples were prepared similarly as in Section 4.9.2, without the addition of AAPH. Concentrations of extracts ranged from 10 to 50 μg/mL. Measurements were made on the Cary Varian Eclipse spectrofluorometer at 37 °C. For DPH and TMA-DPH probes, fluorescence anisotropy (r) was measured based on the following Equation (4). The excitation wavelength was 360 nm, and the emission wavelength was 430 nm [78,88].

(4) $$r=\frac{I_{\|}-GI_{⊥}}{I_{\|}+2GI_{⊥}}$$

where I‖—the intensity of the probe fluorescence is measured vertically, I⊥—the intensity of the probe fluorescence is measured horizontally, and G—correction factor.

### 4.12. Statistical Analysis

Statistical analysis was performed in Excel 2019 (Microsoft, Washington, DC, USA) and Past 4.16c [89] Significant differences were calculated using one-way analysis of variance (ANOVA) with Tukey’s pairwise test marked as * with *p* < 0.05 to the control group. Graphs were prepared in OriginPro 2024 (64-bit) (Northampton, MA, USA).

## 5. Conclusions

Organisms are constantly exposed to stress from the environment such as free radicals. Irregular oxidative homeostasis leads to abnormalities and causes illness. Organisms develop mechanisms that cope with oxidative stress and regulate their homeostasis. However, it is important to know that the membrane of the cells is the first barrier that interacts with the environment, and its abnormalities lead to the death of the cells. Erythrocytes are the cells that are mostly exposed to oxidative stress and are necessary for the normal function of the body. Therefore, it is important to find additional substances that will help organisms combat oxidative stress. Extracts are a great source of polyphenolic compounds that possess antioxidant activities. In this research, we tested and compared the bioactivity of three extracts derived from the leaves of sea buckthorn (BE) as well as the leaves (LE) and roots (RE) of Japanese knotweed. Analyses showed that all the extracts contain a large number of polyphenols, mainly from the group of tannins in BE and a group of procyanidins in RE and LE. Extracts that had high antioxidant activities showed that they protected erythrocytes against oxidative-inductor AAPH. The possible mechanism of their action is that they are mostly localized in the outer part of the membrane, where they can directly protect the cells from the free radicals coming from the environment. Our results showed that sea buckthorn and Japanese knotweed are great plant sources of bioactive and antioxidant compounds. They are not toxic to the erythrocytes and also protect them at a great level from oxidation. Our in vitro studies provide a basis for further research on blood cells and other cells of the circulatory system in the context of the use of extracts as substances against many cardiovascular diseases. In addition, the research can be used for studies on living organisms. Thus, in the future, sea buckthorn and Japanese knotweed extracts could be used as dietary supplements or medicines.

## Figures and Tables

**Table 1:** EC_50_ values (µg/mL) for BE, RE, and LE extracts and standard antioxidant–L (+)-ascorbic acid that caused 50% reduction of the DPPH radical. Statistically significant differences are marked as * with *p* < 0.05.

Extract/Substance	EC_50_ [μg/mL ± SD]
BE	6.28 ± 0.06 *
RE	5.89 ± 0.47 *
LE	6.03 ± 0.65 *
L (+)-ascorbic acid	11.59 ± 0.27

**Table 2:** IC_50_ values (µg/mL) for BE, RE, LE extracts, and Trolox*^®^* that caused 50% inhibition of the AAPH-induced oxidation of erythrocytes and erythrocyte membranes (ghosts) after 90 min.

IC_50_ [μg/mL] ± SD		
Extract/Object	Erythrocytes	Erythrocytes Membrane
BE	13.7 ± 3.07	6.57 ± 1.12
RE	15.7 ± 1.34	6.42 ± 0.68
LE	6.00 ± 3.02	7.55 ± 2.90
AA/Trolox^®^	32.5 ± 4.2 *	3.90 ± 0.60 **

* Results for AA were published before in [26]. ** Results for Trolox^®^ were published before in [27].

## Data Availability

The data presented in this study are available in this article and Supplementary Materials, available online: http://dx.doi.org/10.57755/r9bn-xk57, accessed on 18 June 2024.

## Supplementary Materials

The following supporting information can be downloaded at: https://www.mdpi.com/article/10.3390/molecules29133090/s1, Table S1. UV and MS spectra data of polyphenols and their derivatives in sea buckthorn (*Hippophae rhamnoides* L.) leaves extracts. Figure S1. UPLC-MS chromatogram profile of the sea buckthorn (*Hippophae rhamnoides* L.) leaves at 280 nm. Peak number identification is displayed in Table S1. Table S2. UV and MS spectra data of polyphenols and their derivatives in Japanese knotweed (*Reynoutria japonica Houtt.*) roots and leaves extracts *Rt—retention time, nd—non-identified. Figure S2. UPLC-MS chromatogram profile of the *Reynoutria japonica Houtt*. roots (a) and leaves (b) extracts at 280 nm. Peak number identification is displayed in Table S2. Figure S3. Inhibition of AAPH-induced hemolysis by the sea buckthorn (*Hippophae rhamnoides* L.) (BE) (a), and Japanese knotweed (*Reynoutria japonica Houtt.*) roots (RE) (b) and leaves (LE) (c) extracts. Figure S4. Shapes of unmodified erythrocytes (a), modified with BE (b), RE (c), and LE (d) extracts were observed under optical microscope at 50 μg/mL concentration. Magnifications were 1000×.

## References

[1] Roth G.A., Mensah G.A., Johnson C.O., Addolorato G., Ammirati E., Baddour L.M., Barengo N.C., Beaton A.Z., Benjamin E.J., Benziger C.P. (2020). Global Burden of Cardiovascular Diseases and Risk Factors, 1990–2019. J. Am. Coll. Cardiol..

[2] Rana A., Samtiya M., Dhewa T., Mishra V., Aluko R.E. (2022). Health Benefits of Polyphenols: A Concise Review. J. Food Biochem..

[3] Pogačnik L., Bergant T., Skrt M., Ulrih N.P., Viktorová J., Ruml T. (2020). In Vitro Comparison of the Bioactivities of Japanese and Bohemian Knotweed Ethanol Extracts. Foods.

[4] Patočka J., Navrátilová Z., Ovando M. (2017). Biologically Active Compounds of Knotweed (*Reynoutria* spp.). Mil. Med. Sci. Lett..

[5] Zeb A. (2004). Important Therapeutic Uses of Sea Buckthorn (*Hippophae*): A Review. J. Biol. Sci..

[6] Rousi A. (1971). The Genus *Hippophaë* L. A Taxonomic Study. Ann. Bot. Fenn..

[7] Yang B., Kallio H. (2002). Composition and Physiological Effects of Sea Buckthorn (*Hippophaë*) lipids. Trends Food Sci. Technol..

[8] Yang B., Kallio H.P. (2001). Fatty Acid Composition of Lipids in Sea Buckthorn (*Hippophaë rhamnoides* L.) Berries of Different Origins. J. Agric. Food Chem..

[9] Criste A., Urcan A.C., Bunea A., Furtuna F.R.P., Olah N.K., Madden R.H., Corcionivoschi N. (2020). Phytochemical Composition and Biological Activity of Berries and Leaves from Four Romanian Sea Buckthorn (*Hippophae rhamnoides* L.) Varieties. Molecules.

[10] Guan T.T.Y., Cenkowski S., Hydamaka A. (2006). Effect of Drying on the Nutraceutical Quality of Sea Buckthorn (*Hippophae rhamnoides* L. ssp. sinensis) Leaves. J. Food Sci..

[11] Eccleston C., Baoru Y., Tahvonen R., Kallio H., Rimbach G.H., Minihane A.M. (2002). Effects of an Antioxidant-Rich Juice (*Sea buckthorn*) on Risk Factors for Coronary Heart Disease in Humans. J. Nutr. Biochem..

[12] Süleyman H., Demirezer L., Büyükokuroglu M.E., Akcay M.F., Gepdiremen A., Banoglu Z.N., Göçer F. (2001). Antiulcerogenic Effect of *Hippophae rhamnoides* L.. Phytotherapy Res..

[13] Upadhyay N.K., Kumar R., Siddiqui M.S., Gupta A. (2011). Mechanism of Wound-Healing Activity of *Hippophae rhamnoides* L. Leaf Extract in Experimental Burns. Evid. Based Complement. Altern. Med..

[14] Bailey J.P., Bímová K., Mandák B. (2009). Asexual Spread versus Sexual Reproduction and Evolution in Japanese Knotweed s.l. Sets the Stage for the “Battle of the Clones”. Biol. Invasions.

[15] Liu L.-T., Guo G., Wu M., Zhang W.-G. (2012). The Progress of the Research on Cardio-Vascular Effects and Acting Mechanism of Polydatin. Chin. J. Integr. Med..

[16] Ma Y., Gong X., Mo Y., Wu S. (2016). Polydatin Inhibits the Oxidative Stress-Induced Proliferation of Vascular Smooth Muscle Cells by Activating the eNOS/SIRT1 Pathway. Int. J. Mol. Med..

[17] Alavi M., Farkhondeh T., Aschner M., Samarghandian S. (2021). Resveratrol Mediates Its Anti-Cancer Effects by Nrf2 Signaling Pathway Activation. Cancer Cell Int..

[18] Ko J.-H., Sethi G., Um J.-Y., Shanmugam M.K., Arfuso F., Kumar A.P., Bishayee A., Ahn K.S. (2017). The Role of Resveratrol in Cancer Therapy. Int. J. Mol. Sci..

[19] Leung S.W., Lai J.H., Wu J.C.-C., Tsai Y.-R., Chen Y.-H., Kang S.-J., Chiang Y.-H., Chang C.-F., Chen K.-Y. (2020). Neuroprotective Effects of Emodin against Ischemia/Reperfusion Injury through Activating ERK-1/2 Signaling Pathway. Int. J. Mol. Sci..

[20] Wróbel-Biedrawa D., Grabowska K., Galanty A., Sobolewska D., Podolak I. (2022). A Flavonoid on the Brain: Quercetin as a Potential Therapeutic Agent in Central Nervous System Disorders. Life.

[21] Sharifi-Rad M., Anil Kumar N.V., Zucca P., Varoni E.M., Dini L., Panzarini E., Rajkovic J., Tsouh Fokou P.V., Azzini E., Peluso I. (2020). Lifestyle, Oxidative Stress, and Antioxidants: Back and Forth in the Pathophysiology of Chronic Diseases. Front. Physiol..

[22] Kaźmierczak T., Bonarska-Kujawa D., Męczarska K., Cyboran-Mikołajczyk S., Oszmiański J., Kapusta I. (2023). Analysis of the Polyphenolic Composition of *Vaccinium* L. Extracts and Their Protective Effect on Red Blood Cell Membranes. Membranes.

[23] Colleran B., Lacy S.N., Retamal M.R. (2020). Invasive Japanese Knotweed (*Reynoutria japonica* Houtt.) and Related Knotweeds as Catalysts for Streambank Erosion. River Res. Appl..

[24] Carter B., Curtis T.G.F., Sheehy-Skeffington M.J. (1992). Coastal Dunes: Geomorphology, Ecology and Management for Conservation. Proceedings of the Third European Dune Congress.

[25] Tsuzuku S. (2013). Local Knowledge about Japanese Vegetables and Herbs among People of Japanese Descent in Southwest British Columbia. Ph.D. Thesis.

[26] Cyboran-Mikołajczyk S., Kleszczyńska H., Oszmiański J., Pasławski R. (2019). *Allium ursinum* L. leaves components modified the physico-chemical properties of red blood cells protecting them from the effects of oxidative stress. Acta Pol. Pharm..

[27] Włoch A., Kapusta I., Bielecki K., Oszmiański J., Kleszczyńska H. (2013). Activity of Hawthorn Leaf and Bark Extracts in Relation to Biological Membrane. J. Membr. Biol..

[28] Bessis M. (1972). Red Cell Shapes. An Illustrated Classification and Its Rationale. Nouv. Rev. Fr. Hematol..

[29] Soares S., Brandão E., Guerreiro C., Soares S., Mateus N., de Freitas V. (2020). Tannins in Food: Insights into the Molecular Perception of Astringency and Bitter Taste. Molecules.

[30] Reed J.D. (1995). Nutritional Toxicology of Tannins and Related Polyphenols in Forage Legumes. J. Anim. Sci..

[31] Murdiati T.B., McSweeney C.S., Campbell R.S.F., Stoltz D.S. (1990). Prevention of Hydrolysable Tannin Toxicity in Goats Fed *Clidemia hirta* by Calcium Hydroxide Supplementation. J. Appl. Toxicol..

[32] Hamada S., Yamamoto T., Koga T., McGhee J.R., Michalek S.M., Yamamoto S. (1985). Chemical Properties and Immunobiological Activities of Streptococcal Lipoteichoic Acids. Zentralblatt Für Bakteriol. Mikrobiol. Und Hygiene. Ser. A Med. Microbiol. Infect. Dis. Virol. Parasitol..

[33] Machado T., Pinto A., Pinto M., Leal I., Silva M., Amaral A., Kuster R., Netto-Dossantos K. (2003). In Vitro Activity of Brazilian Medicinal Plants, Naturally Occurring Naphthoquinones and Their Analogues, against Methicillin-Resistant *Staphylococcus aureus*. Int. J. Antimicrob. Agents.

[34] Funatogawa K., Hayashi S., Shimomura H., Yoshida T., Hatano T., Ito H., Hirai Y. (2004). Antibacterial Activity of Hydrolyzable Tannins Derived from Medicinal Plants against *Helicobacter pylori*. Microbiol. Immunol..

[35] Buzzini P., Arapitsas P., Goretti M., Branda E., Turchetti B., Pinelli P., Ieri F., Romani A. (2008). Antimicrobial and Antiviral Activity of Hydrolysable Tannins. Mini-Rev. Med. Chem..

[36] Hagerman A.E., Riedl K.M., Jones G.A., Sovik K.N., Ritchard N.T., Hartzfeld P.W., Riechel T.L. (1998). High Molecular Weight Plant Polyphenolics (Tannins) as Biological Antioxidants. J. Agric. Food Chem..

[37] Tang L., Lv H., Li S., Bi H., Gao X., Zhou J. (2016). Protective Effects of Polyphenol Extracts from Sea Buckthorn (*Hippophaë rhamnoides* L.) on Rat Hearts. Open J. Mol. Integr. Physiol..

[38] Asofiei I., Calinescu I., Trifan A., Gavrila A.I. (2019). A Semi-Continuous Process for Polyphenols Extraction From Sea Buckthorn Leaves. Sci. Rep..

[39] Asofiei I., Calinescu I., Trifan A., David I.G., Gavrila A.I. (2016). Microwave-Assisted Batch Extraction of Polyphenols from Sea Buckthorn Leaves. Chem. Eng. Commun..

[40] Heinäaho M., Pusenius J., Julkunen-Tiitto R. (2006). Effects of Different Organic Farming Methods on the Concentration of Phenolic Compounds in Sea Buckthorn Leaves. J. Agric. Food Chem..

[41] Tsao R. (2010). Chemistry and Biochemistry of Dietary Polyphenols. Nutrients.

[42] van Dijk C., Driessen A.J., Recourt K. (2000). The Uncoupling Efficiency and Affinity of Flavonoids for Vesicles. Biochem. Pharmacol..

[43] Uchida S., Ohta H., Edamatsu R., Hiramatsu M., Mori A., Nonaka G., Nishioka I., Niwa M., Akashi T., Ozaki M. (1988). Active Oxygen Free Radicals are Scavenged by Condensed Tannins. Prog. Clin. Biol. Res..

[44] Cossins E., Lee R., Packer L. (1998). Esr Studies of Vitamin c Regeneration, Order of Reactivity of Natural Source Phytochemical Preparations. IUBMB Life.

[45] Blazsó G., Gábor M., Rohdewald P. (1997). Antiinflammatory Activities of Procyanidin-Containing Extracts from Pinus Pinaster Ait. after Oral and Cutaneous Application. Pharmazie.

[46] Facinó R., Carini M., Aldini G., Berti F., Rossoni G., Bombardelli E., Morazzoni P. (1996). Procyanidines from *Vitis vinifera* Seeds Protect Rabbit Heart from Ischemia/Reperfusion Injury: Antioxidant Intervention and/or Iron and Copper Sequestering Ability. Planta Medica.

[47] Packer L., Rimbach G., Virgili F. (1999). Antioxidant Activity and Biologic Properties of a Procyanidin-Rich Extract from Pine (*Pinus maritima*) Bark, Pycnogenol. Free. Radic. Biol. Med..

[48] Jug U., Naumoska K., Vovk I. (2021). (−)-Epicatechin—An Important Contributor to the Antioxidant Activity of Japanese Knotweed Rhizome Bark Extract as Determined by Antioxidant Activity-Guided Fractionation. Antioxidants.

[49] Chen H., Deng Q., Ji X., Zhou X., Kelly G., Zhang J. (2016). Glucose Oxidase-Assisted Extraction of Resveratrol from Japanese Knotweed (*Fallopia japonica*). New J. Chem..

[50] Bensa M., Glavnik V., Vovk I. (2020). Leaves of Invasive Plants—Japanese, Bohemian and Giant Knotweed—The Promising New Source of Flavan-3-ols and Proanthocyanidins. Plants.

[51] Lamuela-Raventós R.M., Apak R., Capanoglu E., Shahidi F. (2017). Folin-Ciocalteu Method for the Measurement of Total Phenolic Content and Antioxidant Capacity. Measurement of Antioxidant Activity & Capacity.

[52] Pérez M., Dominguez-López I., Lamuela-Raventós R.M. (2023). The Chemistry Behind the Folin–Ciocalteu Method for the Estimation of (Poly)Phenol Content in Food: Total Phenolic Intake in a Mediterranean Dietary Pattern. J. Agric. Food Chem..

[53] Cyboran S., Strugała P., Włoch A., Oszmiański J., Kleszczyńska H. (2015). Concentrated Green Tea Supplement: Biological Activity and Molecular Mechanisms. Life Sci..

[54] Bors W., Heller W., Michel C., Saran M. (1990). Radical Chemistry of Flavonoid Antioxidants. Adv. Exp. Med. Biol..

[55] Platzer M., Kiese S., Herfellner T., Schweiggert-Weisz U., Eisner P. (2021). How Does the Phenol Structure Influence the Results of the Folin-Ciocalteu Assay?. Antioxidants.

[56] Huang D., Ou B., Prior R.L. (2005). The Chemistry behind Antioxidant Capacity Assays. J. Agric. Food Chem..

[57] Everette J.D., Bryant Q.M., Green A.M., Abbey Y.A., Wangila G.W., Walker R.B. (2010). Thorough Study of Reactivity of Various Compound Classes toward the Folin-Ciocalteu Reagent. J. Agric. Food Chem..

[58] Sánchez-Rangel J.C., Benavides J., Heredia J.B., Cisneros-Zevallos L., Jacobo-Velázquez D.A. (2013). The Folin–Ciocalteu Assay Revisited: Improvement of Its Specificity for Total Phenolic Content Determination. Anal. Methods.

[59] Liang N., Kitts D.D. (2014). Antioxidant Property of Coffee Components: Assessment of Methods that Define Mechanisms of Action. Molecules.

[60] Anggraini T., Wilma S., Syukri D., Azima F. (2019). Total Phenolic, Anthocyanin, Catechins, DPPH Radical Scavenging Activity, and Toxicity of *Lepisanthes alata (Blume) Leenh*. Int. J. Food Sci..

[61] Nanjo F., Goto K., Seto R., Suzuki M., Sakai M., Hara Y. (1996). Scavenging Effects of Tea Catechins and Their Derivatives on 1,1-Diphenyl-2-Picrylhydrazyl Radical. Free. Radic. Biol. Med..

[62] Nariya P., Nariya M., Shukla V., Acharya R., Bhalodia N. (2013). In Vitro Evaluation of Antioxidant Activity of Cordia Dichotoma (Forst f.) Bark. Ayu.

[63] Tanwar H., Shweta, Singh D., Singh S.B., Ganju L., Ananth M.K., Narsimhan S. (2017). Anti-Inflammatory Activity of the Functional Groups Present in *Hippophae rhamnoides* (Seabuckthorn) Leaf Extract. Inflammopharmacology.

[64] Upadhyay N.K., Kumar M.Y., Gupta A. (2010). Antioxidant, Cytoprotective and Antibacterial Effects of Sea buckthorn (*Hippophae rhamnoides* L.) Leaves. Food Chem. Toxicol..

[65] Kovářová M., Maděra P., Frantík T., Novák J., Vencl Š. (2022). Effects of Knotweed-Enriched Feed on the Blood Characteristics and Fitness of Horses. Agriculture.

[66] Filho V.M.B., Waczuk E.P., Kamdem J.P., Abolaji A.O., Lacerda S.R., da Costa J.G.M., de Menezes I.R.A., Boligon A.A., Athayde M.L., da Rocha J.B.T. (2014). Phytochemical Constituents, Antioxidant Activity, Cytotoxicity and Osmotic Fragility Effects of Caju (*Anacardium microcarpum*). Ind. Crop. Prod..

[67] Oteiza P. (1994). A Mechanism for the Stimulatory Effect of Aluminum on Iron-Induced Lipid Peroxidation. Arch. Biochem. Biophys..

[68] Kolanjiappan K., Manoharan S., Kayalvizhi M. (2002). Measurement of Erythrocyte Lipids, Lipid Peroxidation, Antioxidants and Osmotic Fragility in Cervical Cancer Patients. Clin. Chim. Acta.

[69] Olchowik E., Lotkowski K., Mavlyanov S., Abdullajanova N., Ionov M., Bryszewska M., Zamaraeva M. (2012). Stabilization of Erythrocytes Against Oxidative and Hypotonic Stress by Tannins Isolated from Sumac Leaves (*Rhus typhina* L.) and Grape Seeds (*Vitis vinifera* L.). Cell. Mol. Biol. Lett..

[70] Cyboran-Mikołajczyk S., Csonka Á., Molnar J., Szabó D., Oszmiański J., Kleszczyńska H. (2018). In Vitro Studies of Anti-Hemolytic and Cytotoxic Activity of Procyanidin-Rich Extract from the Leaves of Actinidia Arguta. Pol. J. Food Nutr. Sci..

[71] Peyrat-Maillard M.N., Cuvelier M.E., Berset C. (2003). Antioxidant Activity of Phenolic Compounds in 2,2′-Azobis (2-Amidinopropane) Dihydrochloride (AAPH)-Induced Oxidation: Synergistic and Antagonistic Effects. J. Am. Oil Chem. Soc..

[72] Puiggròs F., Sala E., Vaqué M., Ardévol A., Blay M., Fernández-Larrea J., Arola L., Bladé C., Pujadas G., Salvadó M.J. (2009). In Vivo, in Vitro, and in Silico Studies of Cu/Zn-Superoxide Dismutase Regulation by Molecules in Grape Seed Procyanidin Extract. J. Agric. Food Chem..

[73] Iglič A., Kralj-Iglič V., Hägerstrand H. (1998). Amphiphile Induced Echinocyte-Spheroechinocyte Transformation of Red Blood Cell Shape. Eur. Biophys. J..

[74] Deuticke B. (1977). Properties and Structural Basis of Simple Diffusion Pathways in the Erythrocyte Membrane. Rev. Physiol. Biochem. Pharmacol..

[75] Gedde M., Huestis W. (1997). Membrane Potential and Human Erythrocyte Shape. Biophys. J..

[76] Virtanen V., Karonen M. (2020). Partition Coefficients (*logP*) of Hydrolysable Tannins. Molecules.

[77] Rue E.A., Rush M.D., van Breemen R.B. (2018). Procyanidins: A Comprehensive Review Encompassing Structure Elucidation via Mass Spectrometry. Phytochem. Rev..

[78] He W. (2023). DPH Probe Method for Liposome-Membrane Fluidity Determination. Methods Mol. Biol..

[79] Bonarska-Kujawa D., Cyboran-Mikołajczyk S., Kleszczyńska H. (2015). Molecular Mechanism of Action of Chlorogenic Acid on Erythrocyte and Lipid Membranes. Mol. Membr. Biol..

[80] Cyboran-Mikołajczyk S., Żyłka R., Jurkiewicz P., Pruchnik H., Oszmiański J., Hof M., Kleszczyńska H. (2017). Interaction of Procyanidin B3 with Membrane Lipids—Fluorescence, DSC and FTIR Studies. Biochim. Biophys. Acta (BBA)—Biomembr..

[81] Gasiorowski K., Szyba K., Brokos B., Kołaczyńska B., Jankowiak-Włodarczyk M., Oszmiański J. (1997). Antimutagenic Activity of Anthocyanins Isolated from Aronia Melanocarpa Fruits. Cancer Lett..

[82] Blainski A., Lopes G.C., De Mello J.C.P. (2013). Application and Analysis of the Folin Ciocalteu Method for the Determination of the Total Phenolic Content from *Limonium brasiliense* L.. Molecules.

[83] Gerçek E., Zengin H., Erişir F.E., Yılmaz Ö. (2021). Biochemical Changes and Antioxidant Capacity of Naringin and Naringenin against Malathion Toxicity in Saccharomyces Cerevisiae. Comp. Biochem. Physiol. Part C Toxicol. Pharmacol..

[84] Cyboran-Mikołajczyk S., Męczarska K., Solarska-Ściuk K., Ratajczak-Wielgomas K., Oszmiański J., Jencova V., Bonarska-Kujawa D. (2022). Protection of Erythrocytes and Microvascular Endothelial Cells against Oxidative Damage by *Fragaria vesca* L. and *Rubus idaeus* L. Leaves Extracts—The Mechanism of Action. Molecules.

[85] Dodge J.T., Mitchell C., Hanahan D.J. (1963). The Preparation and Chemical Characteristics of Hemoglobin-Free Ghosts of Human Erythrocytes. Arch. Biochem. Biophys..

[86] Bradford M.M. (1976). A Rapid and Sensitive Method for the Quantitation of Microgram Quantities of Protein Utilizing the Principle of Protein-Dye Binding. Anal. Biochem..

[87] Męczarska K., Cyboran-Mikołajczyk S., Włoch A., Bonarska-Kujawa D., Oszmiański J., Kleszczynska H. (2017). Polyphenol Content and Bioactivity of Saskatoon (*Amelanchier alnifolia* nutt.) Leaves and Berries. Acta Pol. Pharm..

[88] Kuhry J.-G., Fonteneau P., Duportail G., Maechling C., Laustriat G. (1983). TMA-DPH: A Suitable Fluorescence Polarization Probe for Specific Plasma Membrane Fluidity Studies in Intact Living Cells. Cell Biophys..

[89] Hammer Ø., Harper D.A.T., Ryan P.D. (2001). PAST: Paleontological statistics software package for education and data analysis. Palaeontol. Electron..

