# Supplementary material for: Protective Effect of Polyphenolic Extracts from Hippophae rhamnoides L. and Reynoutria japonica Houtt. on Erythrocyte Membrane
Source: Molecules. 2024 Jun 28;29(13):3090. doi: 10.3390/molecules29133090 (PMC11243633; doi:10.3390/molecules29133090)
Supplement: Supplementary file 1 [file molecules-29-03090-s001.zip › molecules-3040897-supplementary.pdf]

Supplementary materials

Article title: Protective Effect of Polyphenolic Extracts from *Hippophae rhamnoides* L. and *Reynoutria japonica Houtt.* on Erythrocyte Membrane

Journal name: The Journal of Molecular Sciences

Authors: Teresa Kaźmierczak[1], Katarzyna Męczarska[1], Sabina Lachowicz-Wiśniewska[2], Sylwia Cyboran-Mikołajczyk[1], Jan Oszmiański[3], Dorota Bonarska-Kujawa[1]

Affiliation and e-mail address of the corresponding author: Wrocław University of Environmental and Life Sciences, The Faculty of Biotechnology and Food Science, Department of Physics and Biophysics, Norwida Str. 25, 50-375 Wrocław, Poland

Correspondence: teresa.kazmierczak@upwr.edu.pl

Table S1. UV and MS spectra data of polyphenols and their derivatives in sea buckthorn (*Hippophae rhamnoides* L.) leaves extracts.

No.	Compunds	Rt* [min]	Molecular ion [m/z]	Fragments	Content [%]
1	*Catechin-hexoside*	3.34	451	289	0.05
2	*Gallocatechine*	3.43	305		0.09
3	*Pedunculagin isomer*	3.59	783	481. 301	2.78
4	*Catechin-gallocatechin*	3.70	593	305. 289	0.88
5	*Pedunculagin isomer*	3.95	783	633. 609. 301	0.04
6	*Pedunculagin*	4.01	783	478. 431. 385. 301	2.12
7	*(+) - Catechine*	4.05	289		0.34
8	*Pterocarinin A*	4.12	1067	533. 377. 355. 301	0.08
9	*Pterocarinin A izomer*	4.18	1067	1023. 933. 625. 533. 463. 301	2.01
10	*Pterocarinin A izomer*	4.22	1067	1023. 933. 625. 533. 463. 301	**2.64**
11	*Casuarinin*	4.32	935	917. 573. 467. 301	**8.43**
12	*Chebulagic acid*	4.52	953	935. 476. 301	**10.34**
13	*Stachyurin*	4.67	935	771. 633. 446. 301	0.69
14	*HHDP-galloylglucoside Isostrictinin*	4.77	936	633. 595. 467. 377. 301	0.31
15	*Hippophaenin B*	4.80	1041	917. 623. 551. 529. 301	0.37
16	*Hippophaenin C*	4.92	1059	935. 633. 529. 467. 301	0.15
17	*Hippophaenin C*	4.98	1059	935. 633. 529. 467. 301	0.30
18	*Procyanidin dimmer type B*	5.03	577	289	0.50
19	*Galloyl-bis-HHDP-glucose III*	5.24	935	609. 301	1.45
20	*Tellimagrandin I. monomeric*	5.47	785	567. 392. 301	0.28
21	*Ellagitannin*	5.68	1085	1067. 935. 542. 467. 301	**5.83**
22	*Ellagitannin*	6.08	1085	931. 785. 639. 542. 392. 301	**5.20**
23	*Ellagitannin*	6.31	1085	935. 815. 785. 639. 301	**7.52**
24	*Ellagic acid rutinoside*	6.38	609	433. 301	0.95
25	*Quercetin-3-O-rutinoside*	6.42	609	301	0.74
26	*Ellagitannin*	6.59	1085	937. 755. 633. 301	0.58
27	*Myricetin dimethyl ether rutinoside*	6.64	653	345	0.13
28	*Kaempferol-hexoside-rhamnose*	6.70	593	285	0.69
29	*Isorhamnetin-dihexoside*	6.90	623	315	1.16

30	*Isorhamnetin-3-O-rutinoside*	7.03	623	315	0.09
31	*Isorhamnetin-3-O-galactoside*	7.11	477	315	0.28
32	*Isorhamnetin-3-O-glucoside*	7.31	477	315	0.50
33	*Isorhamnetin-3-O-Hexoside-7-O-Rhamnoside*	7.42	623	315	0.04
34	*Isorhamnetin-3-O-rutinoside isomer*	7.55	623	315	0.02
35	*Kaempferol-3-O-rutinoside*	7.75	593	477. 285	0.18
36	*Quercetin-glucoside-rhamnoside-rhamnoside*	7.88	775	612. 301	0.23
37	*Quercetin-hexoside-rhamnoside-rhamnoside*	9.90	775	612. 463. 301	0.02
38	*Isorhamnetion-dihexoside-Hexoside*	10.25	789	626. 477. 315	0.07
39	*Isorhamnetion-dihexoside-Hexoside*	10.74	789	626. 477. 315	0.11
40	*Kaempferol-Hexoside-p-Coumaroil*	10.88	593	447. 285	0.07
	**TOTAL**				**58.26**

*Rt. - retention time.

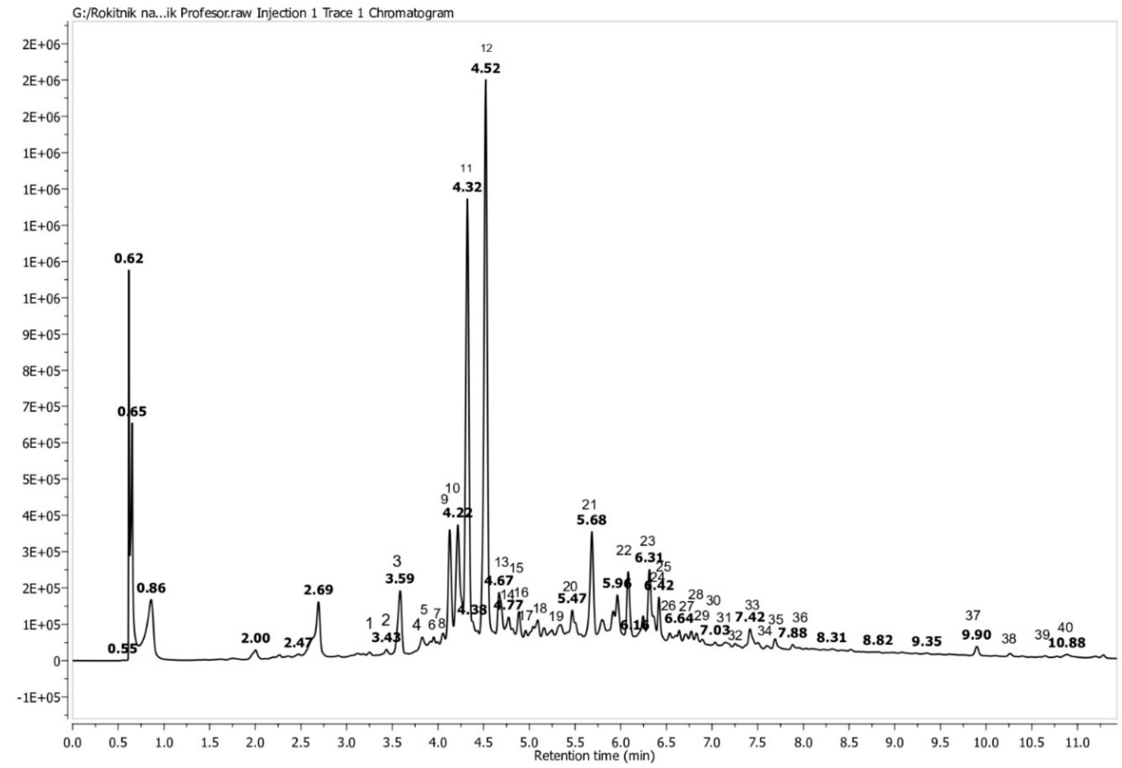

Figure S1. UPLC-MS chromatogram profile of the sea buckthorn (*Hippophae rhamnoides* L.) leaves at 280 nm. Peak number identification is displayed in Table S1.

Table S2. UV and MS spectra data of polyphenols and their derivatives in Japanese knotweed (*Reynoutria japonica Houtt.*) roots and leaves extracts *Rt – retention time, nd – non-identified.

No.	Compounds	Rt. [min]	λ (nm)	Molecular ion [m/z]	Fragments	Content [%]	
						roots	leaves
1	*galloyl glucose*	2.62	277	331	169	0.15	0.02
2	*procyanidin trimmer a*	3.44	279	865	289	0.21	0.01
3	*3-o-caffeoylquinic acid*	3.65	324	353	191	nd*	0.56
4	*procyanidin trimmer a*	3.44	279	865	289	0.21	0.01
5	*procyanidin dimer a*	3.85	279	575	289	0.16	0.01
6	*procyanidin dimer a*	3.91	279	575	289	0.36	0.03
7	*caftaric acid*	4.06	328	311	179	nd	0.03
8	*p-coumaroylquinic acid*	4.20	308	337	163	nd	0.14
9	*p-coumaroylquinic acid*	4.50	309	337	163	nd	0.56
10	*feruloylquinic acid*	4.84	321	367	193/175	nd	0.20
11	*procyanidin dimer b*	4.90	279	577	289	1.34	0.31
12	*procyanidin dimer b*	5.05	279	577	289	0.40	0.34
13	*5-o-caffeoylquinic acid*	5.09	325	353	191	0.00	0.61
14	*(-)-epicatechin*	5.61	279	289		2.64	0.78
15	*procyanidin dimer b*	5.91	279	577	289	2.62	0.96
16	*p-coumaroylquinic acid*	6.34	310	337	191	nd	0.11
17	*procyanidin trimmer a*	6.85	279	865	289	2.09	0.99
18	*astringin*	7.04	319	405	243	0.10	nd
19	*trans-resveratroloside*	7.19	303	449	407/227	1.66	0.01
20	*procyanidinb gallate*	7.49	279	729	289	1.97	0.19
21	*procyanidin tetramer b*	7.73	279	1153	289	0.10	0.10
22	*trans-piceid*	7.92	305	389	227	3.40	0.07
23	*quercetin 3-o-rutinoside*	8.00	350	609	301	nd	0.06
24	*procyanidin tetramer b*	8.09	279	1153	289	0.30	0.20
25	*quercetin 3-o-galactoside*	8.31	350	463	301	nd	0.07
26	*cis-piceid*	8.36	286	389	227	nd	0.01
27	*procyanidin trimmer a*	8.37	279	865	289	0.28	0.15
28	*quercetin 3-o-glucoside*	8.39	350	463	301	nd	0.08
29	*(+)-catechin gallate*	8.62	279	441	289	0.73	0.05
30	*quercetin pentoside*	8.91	350	433	301	nd	0.07
31	*quercetin pentoside*	8.99	350	433	301	nd	0.23
32	*quercetin pentoside*	9.06	350	433	301	nd	0.07
33	*resveratroloside*	9.26	322	449	389/227	0.29	nd
34	*quercetin rhamnoside*	9.34	347	447	301	nd	3.51
35	*resveratrol*	11.18	305	227		0.56	nd
	procyanidin polymers					21.33	59.74
TOTAL						**40.90**	**70.28**

(a)

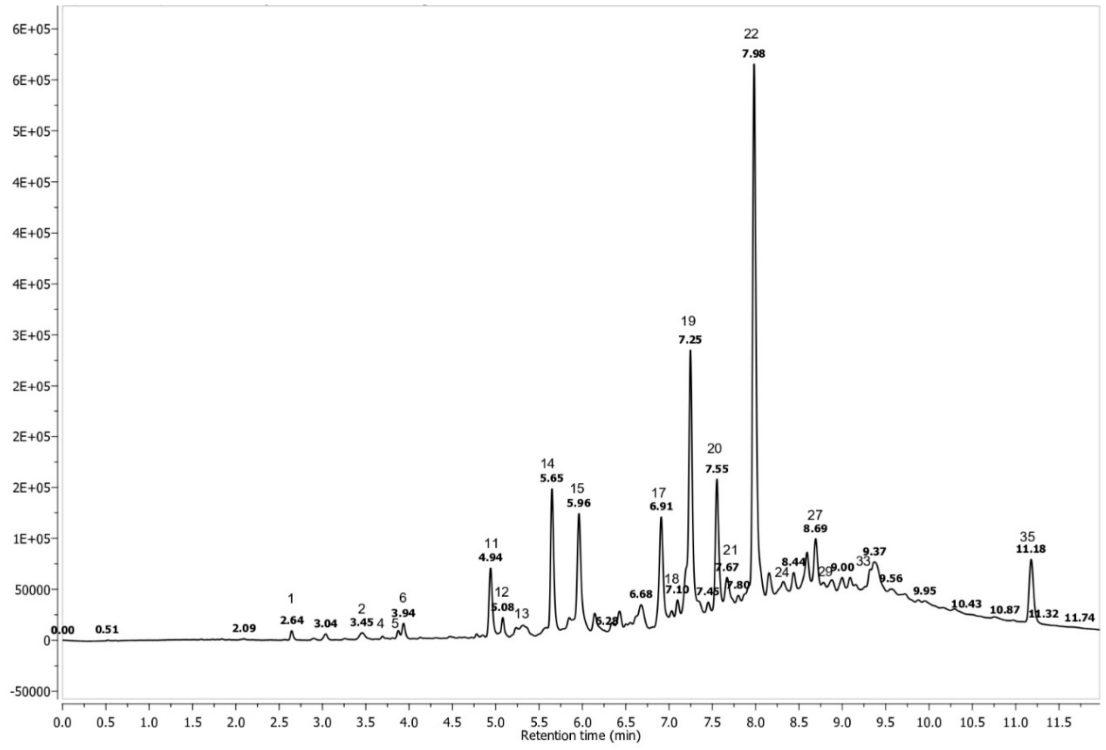

(b)

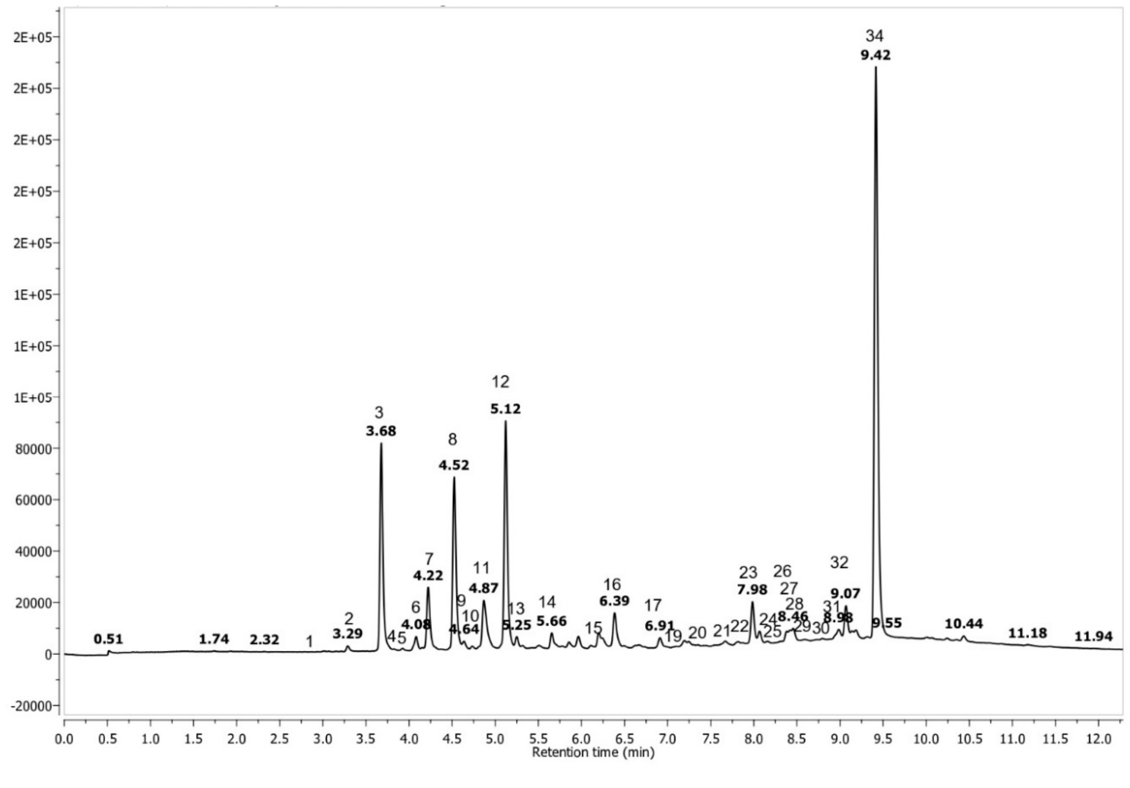

Figure S2. UPLC-MS chromatogram profile of the *Reynoutria japonica Houtt.* roots **(a)** and leaves **(b)** extracts at 280 nm. Peak number identification is displayed in Table S2.

Inhibition of the hemolysis induced by AAPH by extracts

(a)

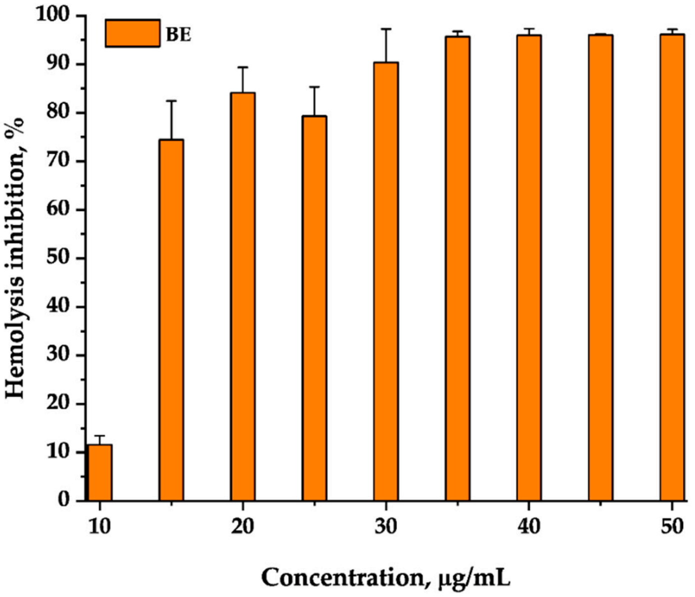

BE
Hemolysis inhibition, %
0
10
20
30
40
50
60
70
80
90
100
10
20
30
40
50
Concentration, μg/mL

(b)

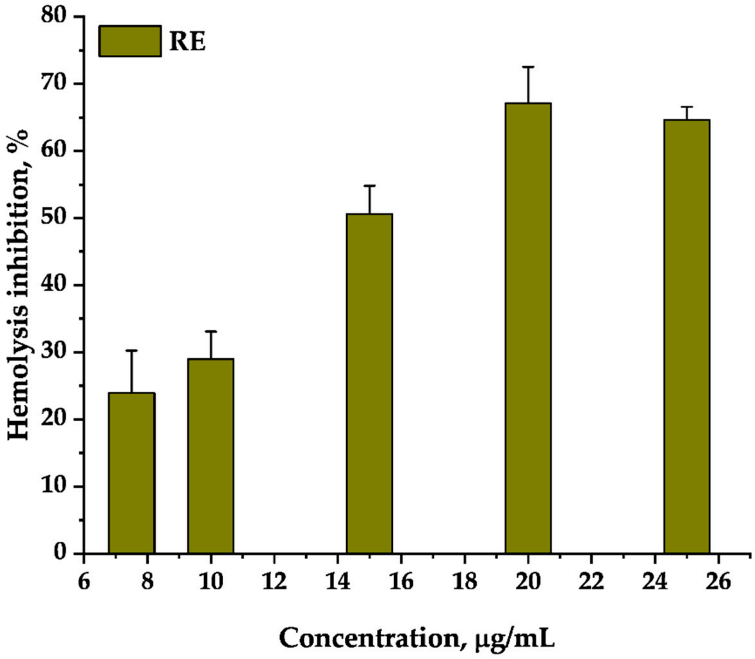

RE
Hemolysis inhibition, %
0
10
20
30
40
50
60
70
80
6
8
10
12
14
16
18
20
22
24
26
Concentration, μg/mL

(c)

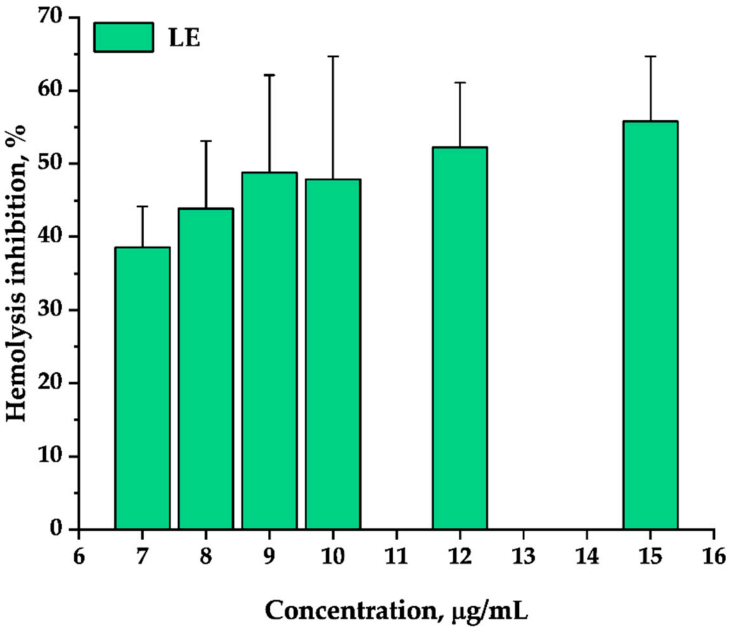

Figure S3. Inhibition of AAPH-induced hemolysis by the sea buckthorn (*Hippophae rhamnoides* L.) (BE) (**a**), and Japanese knotweed (*Reynoutria japonica Houtt.*) roots (RE) (**b**) and leaves (LE) (**c**) extracts.

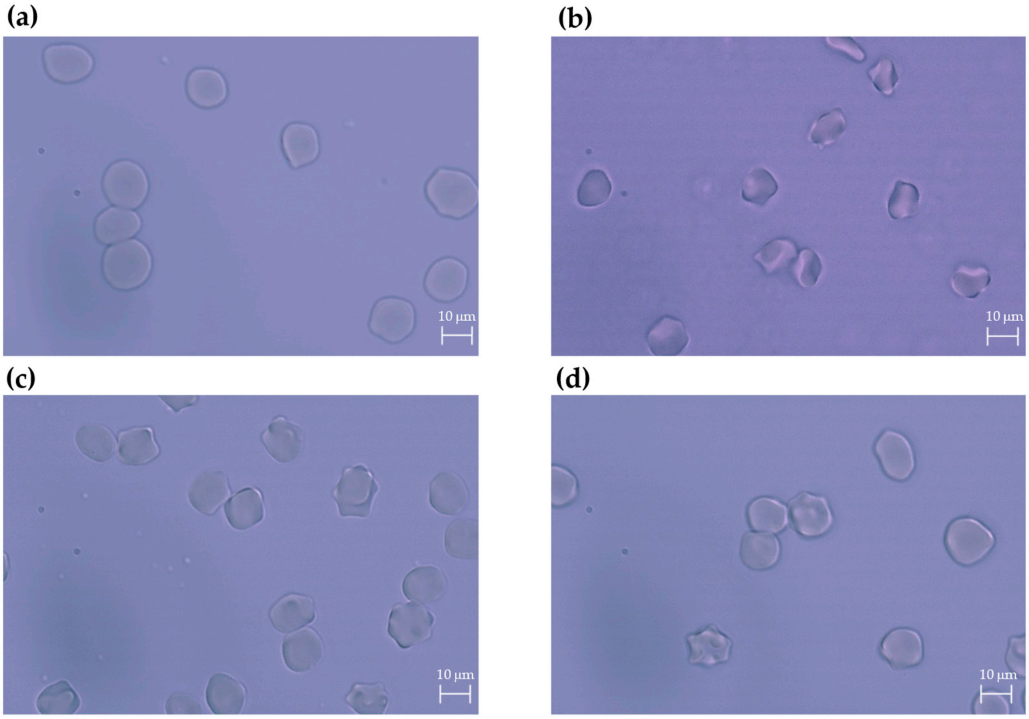

Figure S4. Shapes of unmodified erythrocytes (**a**), modified with BE (**b**), RE (**c**), and LE (**d**) extracts were observed under optical microscope at 50 µg/mL concentration. Magnifications were 1000x.
